# Supplementary material for: Novel Driver Strength Index highlights important cancer genes in TCGA PanCanAtlas patients
Source: PeerJ. 2022 Aug 11;10:e13860. doi: 10.7717/peerj.13860 (PMC9375969; doi:10.7717/peerj.13860)
Supplement: Supplemental Information 2 [file peerj-10-13860-s002.zip › COHORTS/genes plots/cohorts/2021_8_16_14_9_distribution_cohorts_females_COAD.pdf]

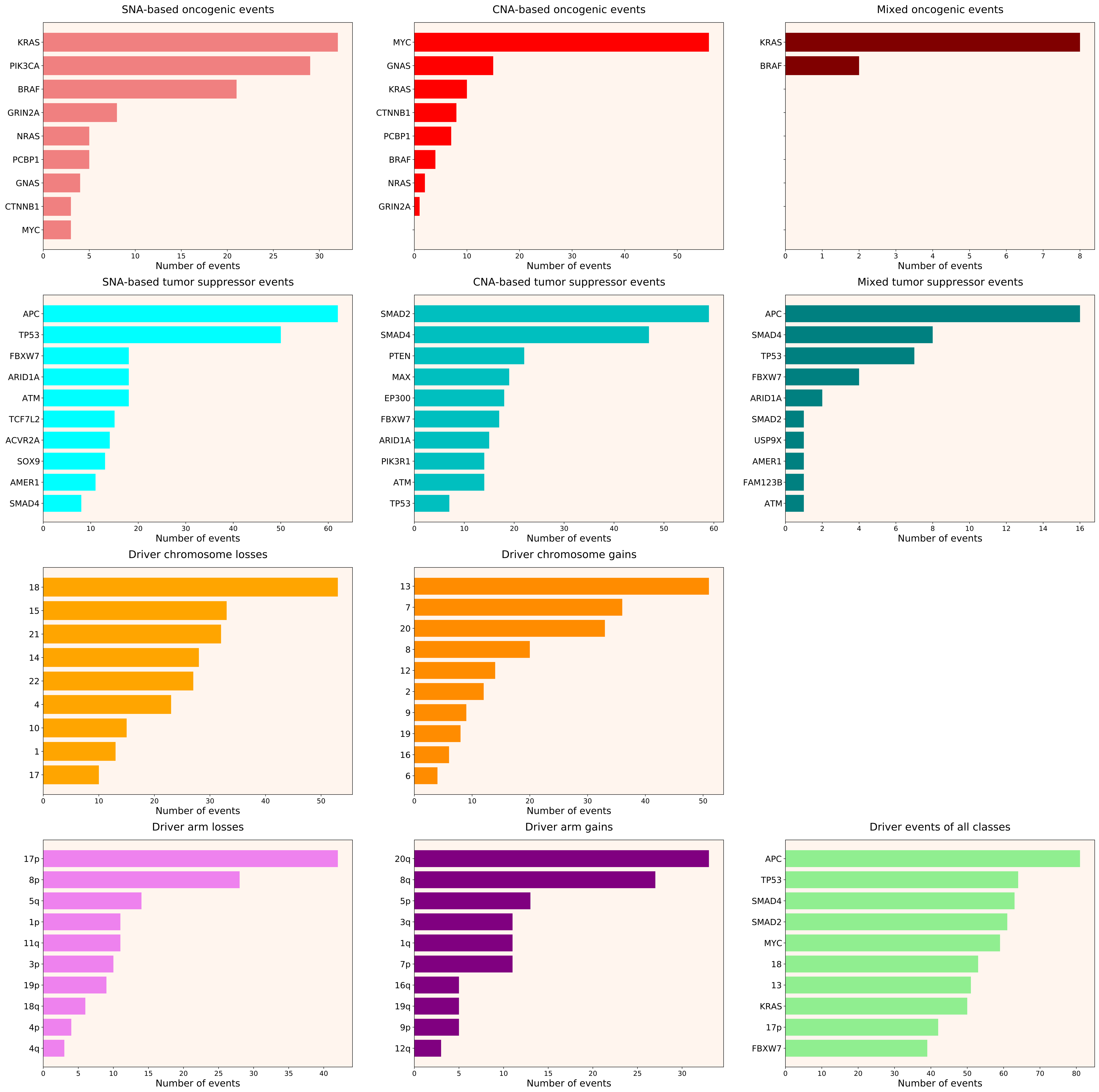
SNA-based oncogenic events
KRAS
PIK3CA
BRAF
GRIN2A
NRAS
PCBP1
GNAS
CTNNB1
MYC
Number of events
CNA-based oncogenic events
MYC
GNAS
KRAS
CTNNB1
PCBP1
BRAF
NRAS
GRIN2A
Number of events
Mixed oncogenic events
KRAS
BRAF
Number of events
SNA-based tumor suppressor events
APC
TP53
FBXW7
ARID1A
ATM
TCF7L2
ACVR2A
SOX9
AMER1
SMAD4
Number of events
CNA-based tumor suppressor events
SMAD2
SMAD4
PTEN
MAX
EP300
FBXW7
ARID1A
PIK3R1
ATM
TP53
Number of events
Mixed tumor suppressor events
APC
SMAD4
TP53
FBXW7
ARID1A
SMAD2
USP9X
AMER1
FAM123B
ATM
Number of events
Driver chromosome losses
18
15
21
14
22
4
10
1
17
Number of events
Driver chromosome gains
13
7
20
8
12
2
9
19
16
6
Number of events
Driver arm losses
17p
8p
5q
1p
11q
3p
19p
18q
4p
4q
Number of events
Driver arm gains
20q
8q
5p
3q
1q
7p
16q
19q
9p
12q
Number of events
Driver events of all classes
APC
TP53
SMAD4
SMAD2
MYC
18
13
KRAS
17p
FBXW7
Number of events
